# Key mRNAs and lncRNAs of pituitary that affect the reproduction of *FecB* + + small tail han sheep

**DOI:** 10.1186/s12864-024-10191-8

**Published:** 2024-04-22

**Authors:** Jianqi Yang, Jishun Tang, Xiaoyun He, Ran Di, Xiaosheng Zhang, Jinlong Zhang, Xiaofei Guo, Wenping Hu, Mingxing Chu

**Affiliations:** 1 State Key Laboratory of Animal Biotech Breeding, Institute of Animal Science, Chinese Academy of Agricultural Sciences (CAAS), 100193 Beijing, China; 2 Institute of Animal Husbandry and Veterinary Medicine, Anhui Academy of Agricultural Sciences, 230031 Hefei, China; 3 Tianjin Key Laboratory of Animal Molecular Breeding and Biotechnology, Tianjin Engineering Research Center of Animal Healthy Farming, Institute of Animal Science and Veterinary, Tianjin Academy of Agricultural Sciences, 300381 Tianjin, China

**Keywords:** Pituitary, mRNAs, lncRNAs, Reproduction, Sheep

## Abstract

**Background:**

The pituitary directly regulates the reproductive process through follicle-stimulating hormone (FSH) and luteinizing hormone (LH). Transcriptomic research on the pituitaries of ewes with different *FecB* (fecundity Booroola) genotypes has shown that some key genes and lncRNAs play an important role in pituitary function and sheep fecundity. Our previous study found that ewes with *FecB* + + genotypes (without *FecB* mutation) still had individuals with more than one offspring per birth. It is hoped to analyze this phenomenon from the perspective of the pituitary transcriptome.

**Results:**

The 12 Small Tail Han Sheep were equally divided into polytocous sheep in the follicular phase (PF), polytocous sheep in the luteal phase (PL), monotocous sheep in the follicular phase (MF), and monotocous sheep in the luteal phase (ML). Pituitary tissues were collected after estrus synchronous treatment for transcriptomic analysis. A total of 384 differentially expressed genes (DEGs) (182 in PF vs. MF and 202 in PL vs. ML) and 844 differentially expressed lncRNAs (DELs) (427 in PF vs. MF and 417 in PL vs. ML) were obtained from the polytocous-monotocous comparison groups in the two phases. Functional enrichment analysis showed that the DEGs in the two phases were enriched in signaling pathways known to play an important role in sheep fecundity, such as calcium ion binding and cAMP signaling pathways. A total of 1322 target relationship pairs (551 pairs in PF vs. MF and 771 pairs in PL vs. ML) were obtained for the target genes prediction of DELs, of which 29 DEL-DEG target relationship pairs (nine pairs in PF vs. MF and twenty pairs in PL vs. ML). In addition, the competing endogenous RNA (ceRNA) networks were constructed to explore the regulatory relationships of DEGs, and some important regulatory relationship pairs were obtained.

**Conclusion:**

According to the analysis results, we hypothesized that the pituitary first receives steroid hormone signals from the ovary and uterus and that *VAV3* (Vav Guanine Nucleotide Exchange Factor 3), *GABRG1* (Gamma-Aminobutyric Acid A Receptor, Gamma 1), and *FNDC1* (Fibronectin Type III Domain Containing 1) played an important role in this process. Subsequently, the reproductive process was regulated by gonadotropins, and *IGFBP1* (Insulin-like Growth Factor Binding Protein 1) was directly involved in this process, ultimately affecting litter size. In addition, *TGIF1* (Transforming Growth Factor-Beta-Induced Factor 1) and *TMEFF2* (Transmembrane Protein With EGF Like And Two Follistatin Like Domains 2) compensated for the effect of the *FecB* mutation and function by acting on TGF-β/SMAD signaling pathway, an important pathway for sheep reproduction. These results provided a reference for understanding the mechanism of multiple births in Small Tail Han Sheep without *FecB* mutation.

**Supplementary Information:**

The online version contains supplementary material available at 10.1186/s12864-024-10191-8.

## Introduction

Larger litter size will not only provide more animal products but also bring more income to breeders, so litter size has always been an important economic trait in sheep breeding [1]. *FecB*, as the main gene for multiple lambs in sheep, was first discovered in Booroola Merino sheep and was subsequently mapped to the *BMPR1B* (Bone Morphogenetic Protein Receptor Type 1) gene on chromosome six by three research groups [2–4]. Small Tail Han Sheep are widely bred for their high fecundity, and many studies on this breed have shown the important role of *FecB* in their high fecundity phenotype [5]. The *FecB* gene was shown to have an additive effect on ovulation rate, and the effect on litter size varied by genotype [6–8]. Compared with *FecB* + + individuals, ewes carrying one copy of the mutation have 1.3–1.6 more ovulations and 0.9–1.2 more lambs, respectively, and ewes carrying two copies of the mutation have 2.7-3.0 more ovulations and 1.1–1.7 more lambs [9–12]. However, our previous research found that in the Small Tail Han Sheep population without *FecB* mutation, some individuals also exhibit multiple lambing trait [13, 14]. We are interested in the mechanism by which this phenomenon occurs.

The reproductive process is affected by multiple organs and multiple hormones [15]. Many studies have tried to interpret the effect of *FecB* on ovulation numbers from the perspective of hormone regulation [16, 17]. Reports have shown that the *FecB* gene was associated with higher concentrations of FSH in peripheral blood during estrus [18–20]. In the study on the reproductive phenotype of Small Tail Han sheep, it was found that there were significant differences in the concentration of FSH and estrogen in the serum of three *FecB* genotype ewes at certain points in the estrus cycle, and the concentration of FSH in *FecB* BB ewes after estrus was higher than that of *FecB* + + ewe [21]. The pituitary gland receives gonadotropin-releasing hormone (GnRH) signals from the hypothalamus, and the LH and FSH are released into the circulation system to promote gonadal growth, gametogenesis, and gonadal hormone release. In other words, the pituitary is the bridge between the hypothalamus and the gonads and plays an important role in the maintenance of reproductive processes and life activities [22]. Given the importance of FSH and LH secreted by the pituitary in reproductive processes, the researchers focused on the pituitary. In the study of pituitary transcriptomics of BB ewes and + + ewes, some genes affecting hormone regulation and the lncRNA-Gene regulatory relationship involving key genes were found, such as LOC105613905 trans-regulated *TGFB1* (Transforming Growth Factor Beta 1) [23].

Rapidly developing advanced RNA-sequencing technologies can provide in-depth analysis of traditional coding RNAs as well as non-coding RNAs [24]. Many important genes related to the reproductive process and their regulatory relationships have been discovered by sequencing technology [25, 26]. lncRNA SM2 participates in the regulation of gonadotropin secretion in sheep pituitary cells by targeting oar-miR-16b/TGF-β/SMAD2 [27]. Through high-throughput sequencing and in vitro validation in sheep pituitary cells, the researchers explored the interaction mode of candidate lncRNA TCONS_00066406 and its target gene *HSD17B12* (Hydroxysteroid 17-Beta Dehydrogenase 12), revealing the potential role of lncRNA in male reproduction [28]. These studies demonstrate the potential of long non-coding RNAs (lncRNAs) as regulators of reproductive processes, as well as the rich mode of action of lncRNAs as regulators [29].

In summary, our focus is on the high-fecundity individuals in the Small Tail Han Sheep population without *FecB* mutation. We hope to compare the differences in the pituitary transcriptome between polytocous and monotocous Small Tail Han Sheep with *FecB* ++. Looking for some genes that affect its high fecundity at the pituitary level, as well as the lncRNA-mRNA regulatory relationships. This work will provide new insights into the mechanism of high fecundity of *FecB* + + Small Tail Han Sheep.

## Results

### Overview of the sequencing data

**Fig. 1:**
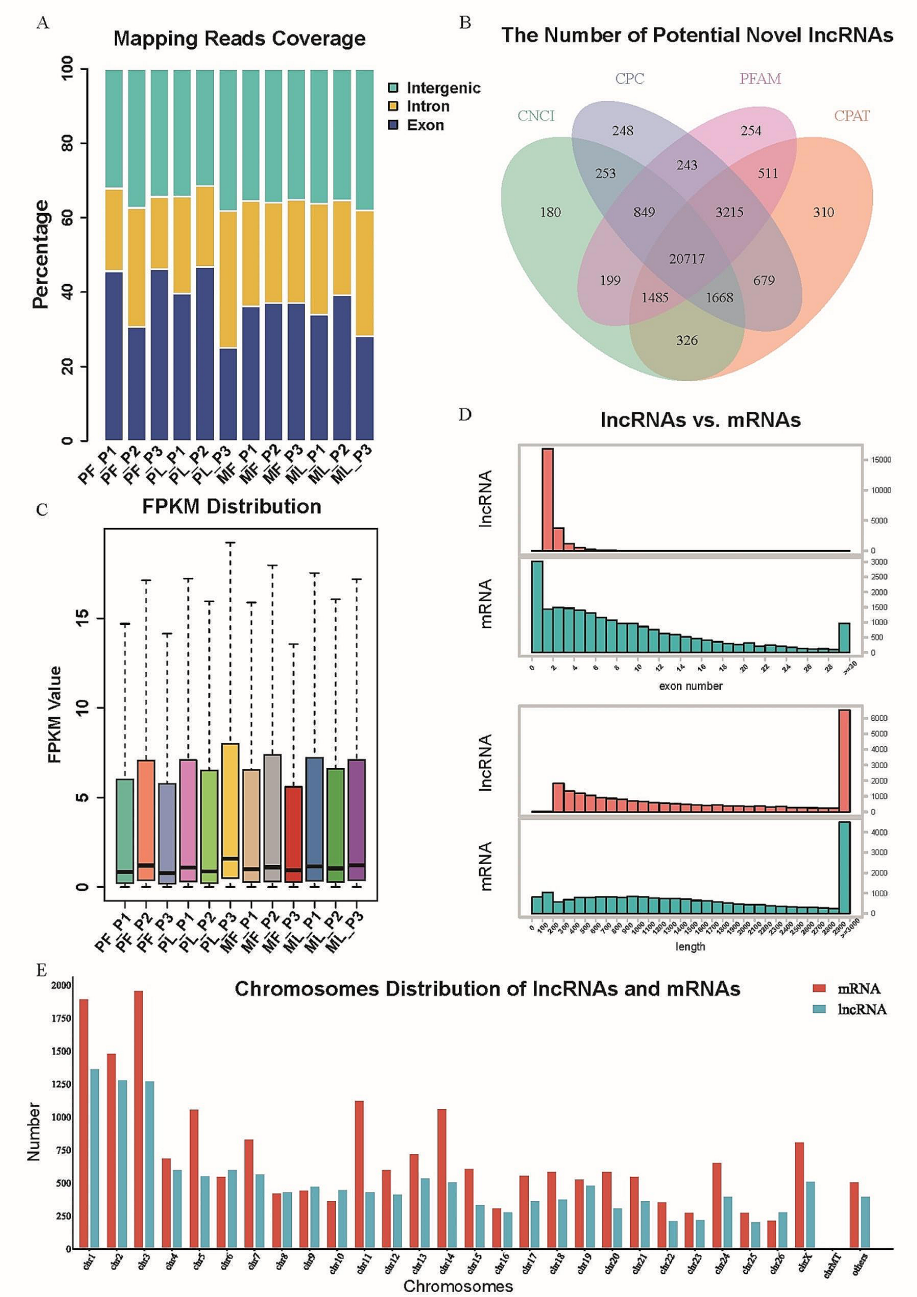
Overview of sequencing results. (**A**) Classification of the uniquely mapped read locations, including exon, intron, and intergenic regions. (**B**) Venn diagram shows the number of novel lncRNAs obtained by four coding potential prediction software. (**C**) The boxplot shows the overall distribution trend of the expression level of the samples. (**D**) Exon number and length characteristics of mRNAs and lncRNAs. (**E**) Distribution of mRNAs and lncRNAs on chromosomes

A total of 1,451,839,480 raw reads were obtained in 12 pituitary samples, with an average of 120,986,623 raw reads per sample. The mapping rate of each sample was above 93%, and the multimap rate of each sample was lower than 6% (Supplementary Table S1). We counted the ratio of the number of sequences uniquely aligned to exon, intron, and intergenic of the reference genome. The sequences aligned to the respective regions averaged 37.1% (exon), 27.5% (intron), and 35.4% (intergenic) (Fig. 1A). In addition to known genes and lncRNAs, we also predicted a total of 20,717 new lncRNAs using Coding-Noncoding Index (CNCI) [30], Coding Potential Calculator (CPC) [31], Protein families database (PFAM) [32], and Coding Potential Assessment Tool (CPAT) [33] (Fig. 1B). From the perspective of expression, we checked the overall distribution trend of the expression of the samples and the respective expression of mRNA and lncRNA in each sample. Overall expression levels were similar between samples, whereas lncRNAs were less abundant than mRNAs in each sample (Fig. 1C and Supplementary Figure S1). In addition, mRNAs had more abundant exon features and length features than lncRNAs (Fig. 1D). As the largest three of the sheep chromosomes, chromosome one, chromosome two, and chromosome three were also the top three chromosomes for mRNA and lncRNA sources (Fig. 1E).

### Profiling of DELs and DEGs in small tail Han sheep pituitary

**Fig. 2:**
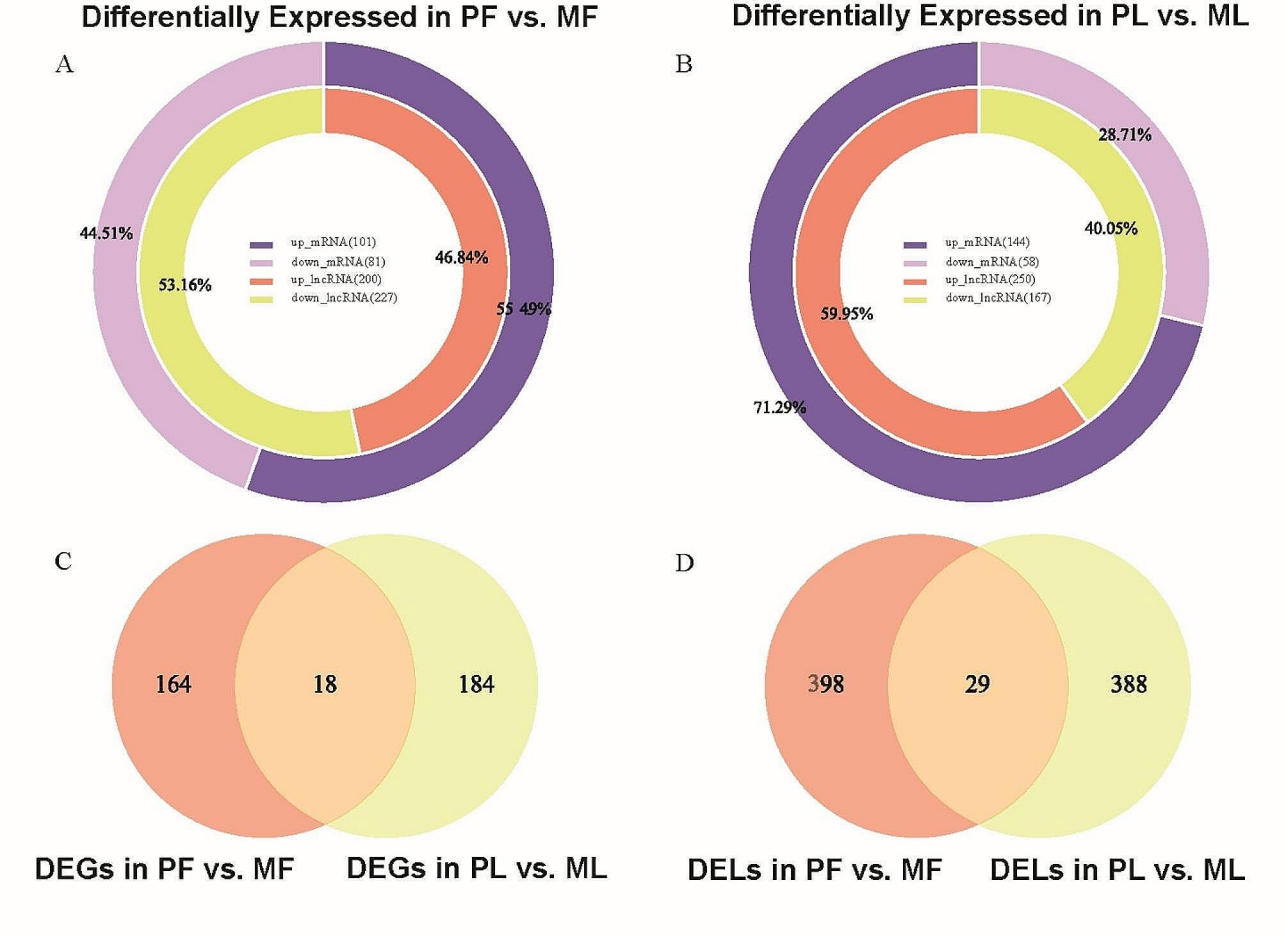
The analysis of DEGs and DELs. (**A**) The number of DEGs and DELs in PF vs. MF, the proportion of up-regulation and down-regulation to the total. (**B**)The number of DEGs and DELs in PL vs. ML, the proportion of up-regulation and down-regulation to the total. (**C**) The overlapped DEGs were differentially expressed in the comparison groups of two periods. (**D**) The overlapped DELs were differentially expressed in the comparison groups of two periods

**Fig. 3:**
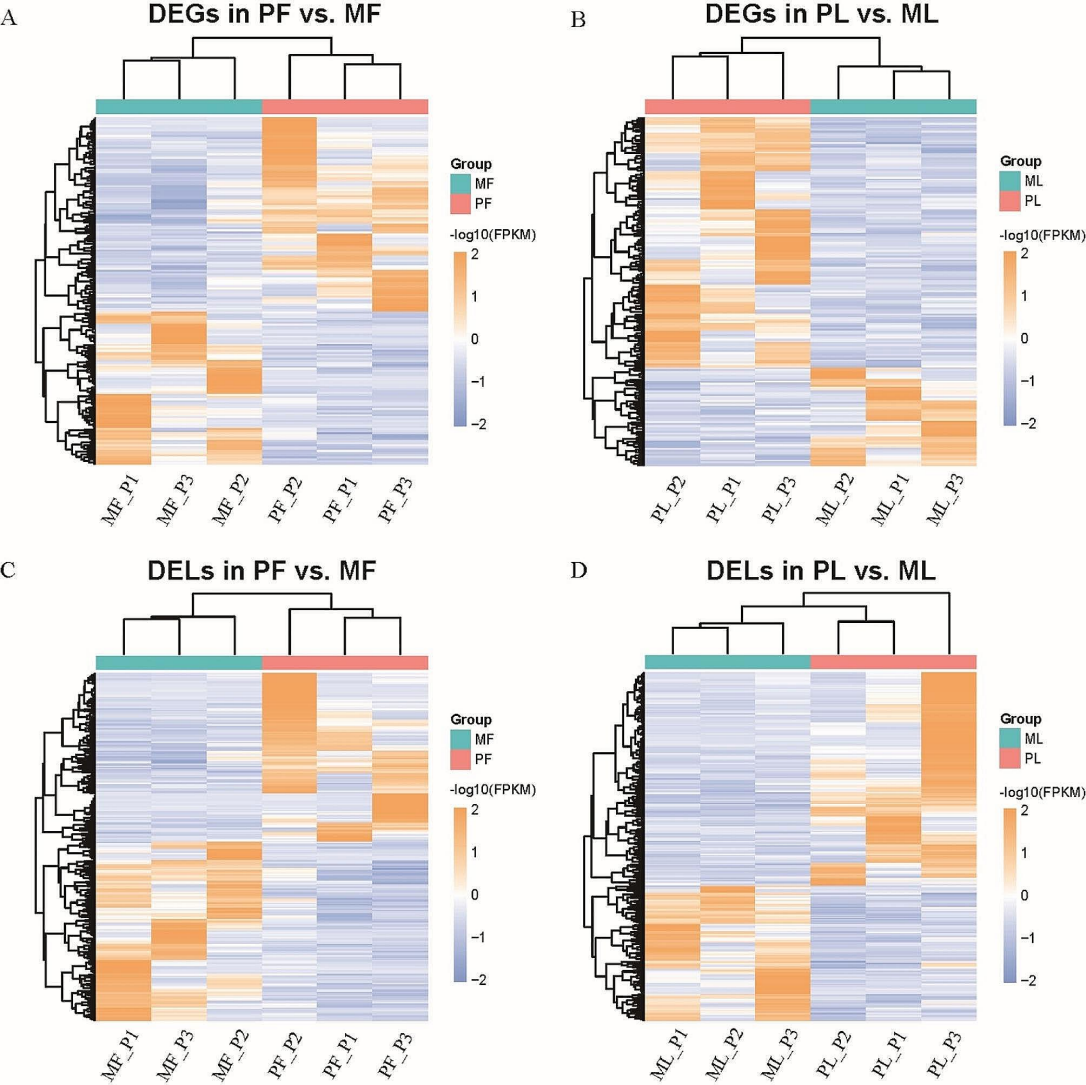
Hierarchical clusters of DEGs and DELs. The heatmaps show the hierarchical clustering results of DEGs and DELs in each group: DEGs in PF vs. MF (**A**), DEGs in PL vs. ML (**B**), DELs in PF vs. MF (**C**), and DELs in PL vs. ML (**D**)

Under the condition of fold change > 1.6 and *P* < 0.05, a total of 384 DEGs (182 in PF vs. MF and 202 in PL vs. ML) (Supplementary Table S2) and 844 DELs (427 in PF vs. MF and 417 in PL vs. ML) (Supplementary Table S3) were selected (Fig. 2A, B). Based on the total number of differences, whether DEGs or DELs, there was not a big gap between the two periods. However, the PL vs. ML had more upregulated DEGs (number = 144, 71.29% of all DEGs). Under more stringent screening conditions (fold change > 2 and *P*_adj_ < 0.05), the sixteen DEGs (down: seven; up: nine) and twenty-five DELs (down: nine; up: sixteen) were identified in PF vs. MF, the eight DEGs (down: two; up: six) and fifteen DELs (down: three; up: twelve) were identified in PL vs. ML. We investigated the functions of these DEGs one by one and noticed a few DEGs (PF vs. MF: *IGFBP1*, *GABRG1*; PL vs. ML: *FNDC1*, *TMEFF2*) that may have an impact on the fertility of the research samples. The heatmaps visualized the clustering results between different libraries. The expression patterns among individuals within a group were similar, and their distances were close, while the expression patterns between groups were different, and the distances were large (Fig. 3A-D).

We also counted the overlapped mRNAs and lncRNAs that were differentially expressed in the comparison groups of two periods (Fig. 2C, D and Supplementary Table S4). Among the eighteen overlapping DEGs, the expression trends of the three DEGs were opposite in the two periods, and the rest were the same. Among the twenty-nine overlapping DELs, the expression trends of fourteen DELs were opposite in the two periods, and the rest were the same. Similarly, by retrieving the functions of these DEGs, we focused on two potentially important genes: *IGFBP1* and *VAV3*.

**Fig. 4:**
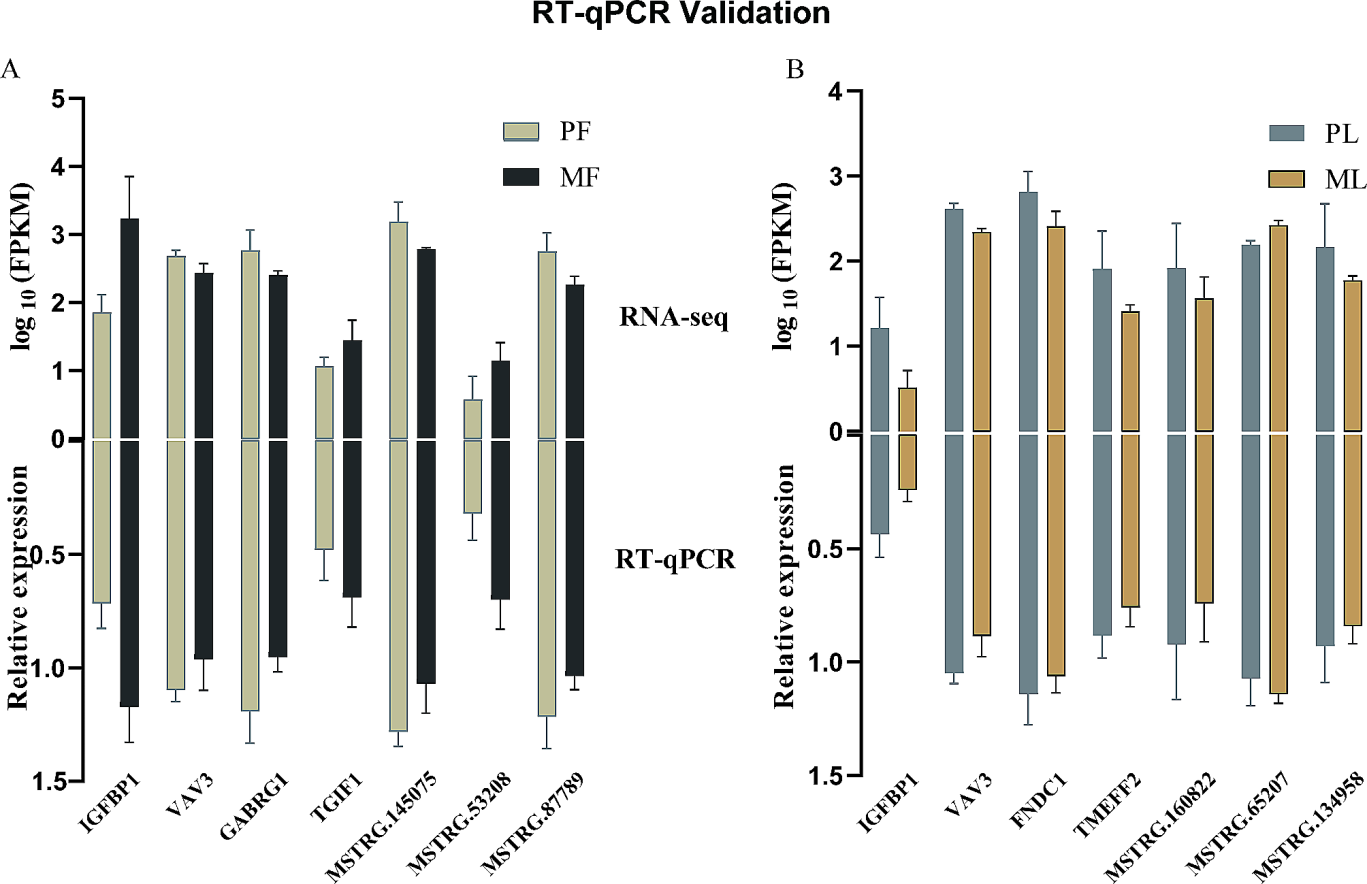
RT-qPCR Validation. (**A**, **C**), RNA-seq results of seven selected mRNAs and lncRNAs in PF vs. MF and PL vs. ML. (**B**, **D**), RT-qPCR results of seven selected mRNAs and lncRNAs in PF vs. MF and PL vs. ML

The results of real-time quantitative polymerase chain reaction (RT-qPCR) verified the accuracy of the sequencing results, both mRNA and lncRNA in the pituitary showed similar expression patterns to the sequencing results (Fig. 4).

### GO analysis of DEGs

**Fig. 5:**
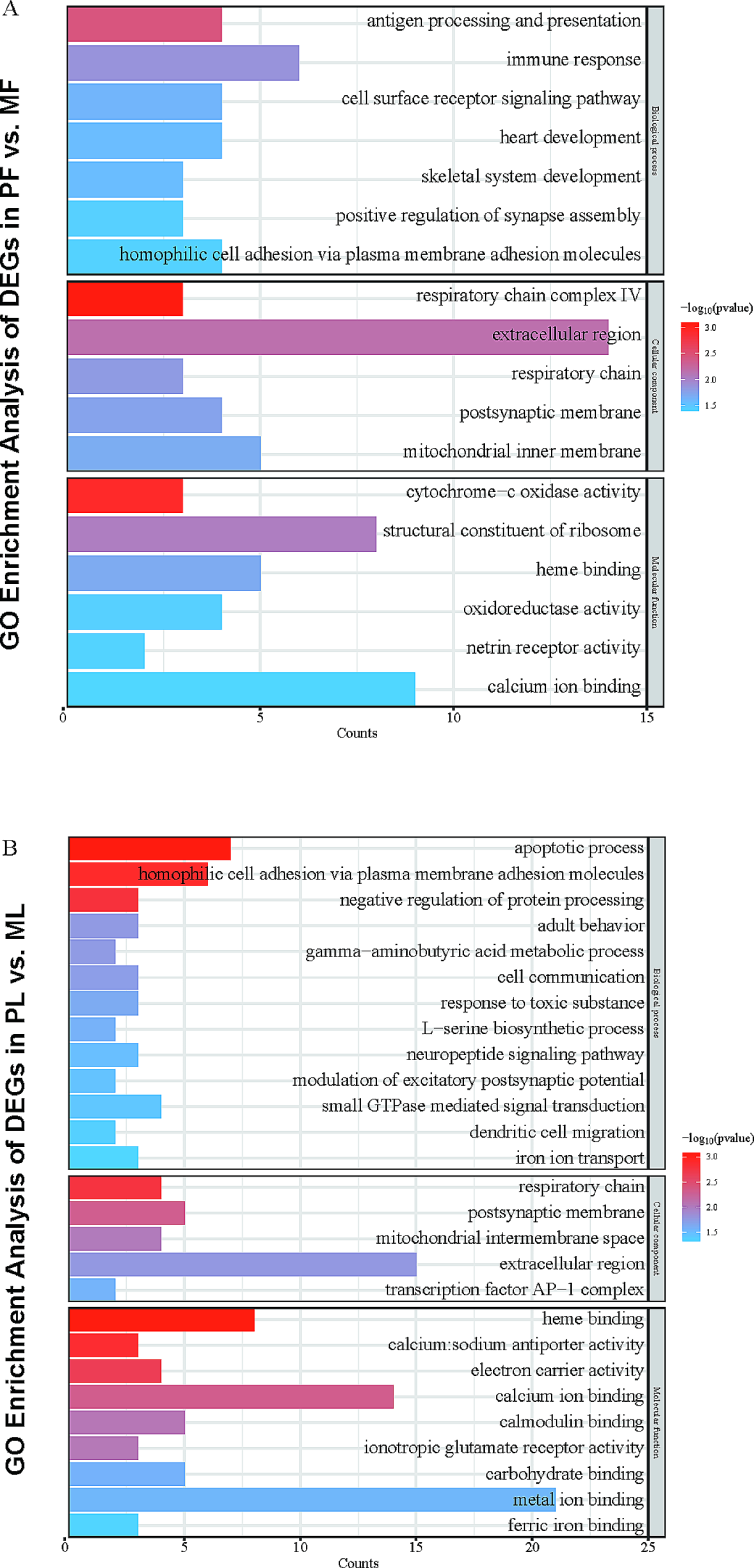
GO analyses of DEGs. DAVID-based GO analyses of DEGs in PF vs. MF (**A**) and PL vs. ML (**B**). Only significantly enriched entries are shown in the figure

To characterize the functions of genes, Gene Ontology (GO) (Supplementary Table S5) analysis was performed using the online analysis tool DAVID [34]. The threshold *P* < 0.05 was set as the screening condition, and only the significantly enriched entries were shown in the figure. A total of eighteen GO items (Fig. 5A) were obtained in PF vs. MF, and a total of twenty-seven GO items (Fig. 5B) in PL vs. ML. The two periods were co-enriched to six GO entries (homophilic cell adhesion via plasma membrane adhesion molecules, extracellular region, respiratory chain, postsynaptic membrane, heme binding, calcium ion binding).

### KEGG analysis of DEGs

**Fig. 6:**
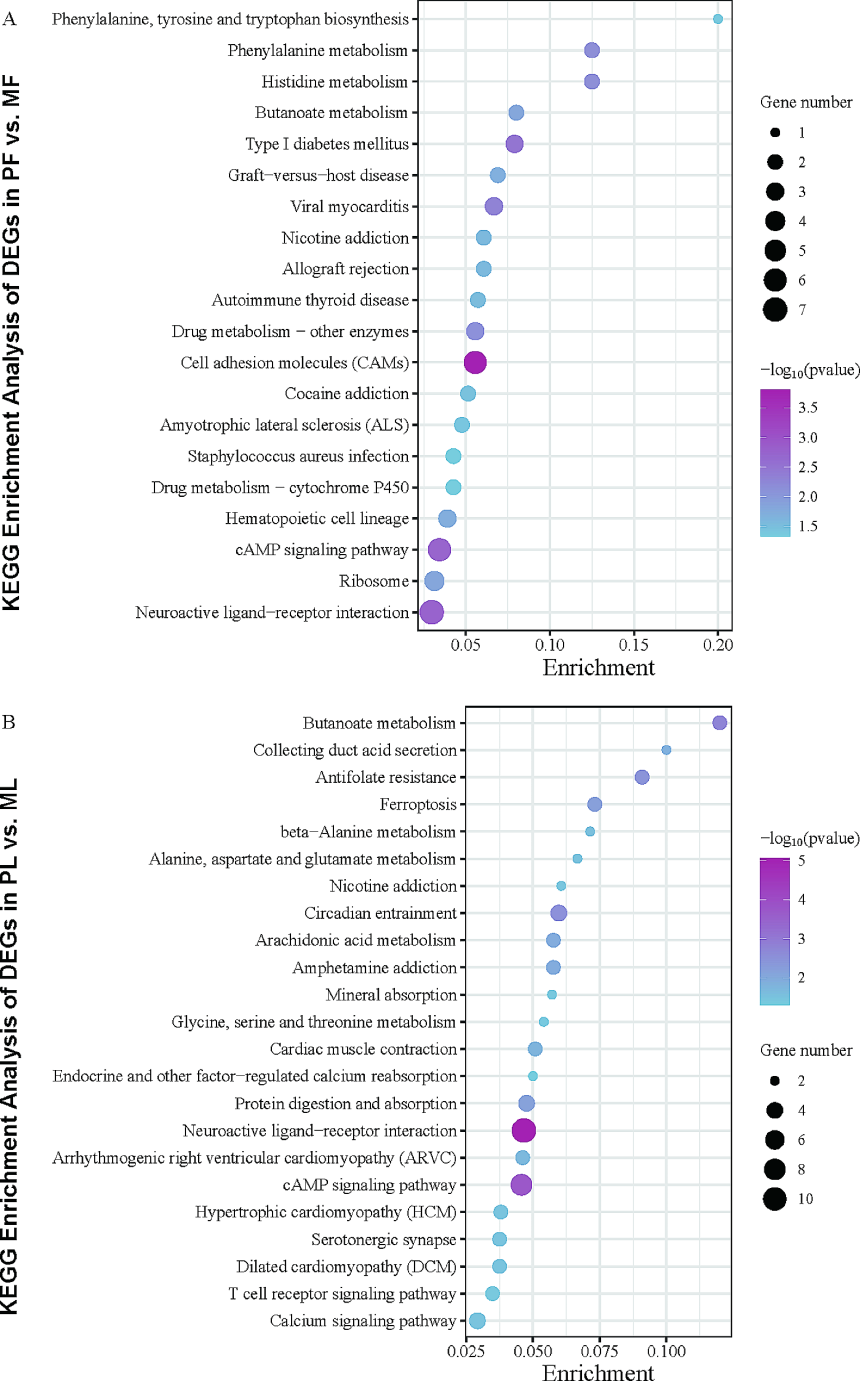
KEGG analyses of DEGs. KOBAS-based KEGG analyses of DEGs in PF vs. MF (**A**) and PL vs. ML (**B**). Only significantly enriched entries are shown in the figure

Kyoto Encyclopedia of Genes and Genomes (KEGG) (Supplementary Table S6) analysis was performed using the online tool KOBAS 3.0 [35]. A total of twenty KEGG items (Fig. 6A) were obtained in PF vs. MF, and a total of twenty-three KEGG items (Fig. 6B) in PL vs. ML. The two periods were co-enriched to four KEGG entries (neuroactive ligand-receptor interaction, cAMP signaling pathway, butanoate metabolism, nicotine addiction). We focused on the two KEGG items: neuroactive ligand-receptor interaction and cAMP signaling pathway which have been shown to have important effects on sheep fecundity. Regardless of sorting all significantly enriched entries by *P-value* or by the number of DEGs included, these two pathways ranked the top in both periods.

### PPI network analysis of DEGs

**Fig. 7:**
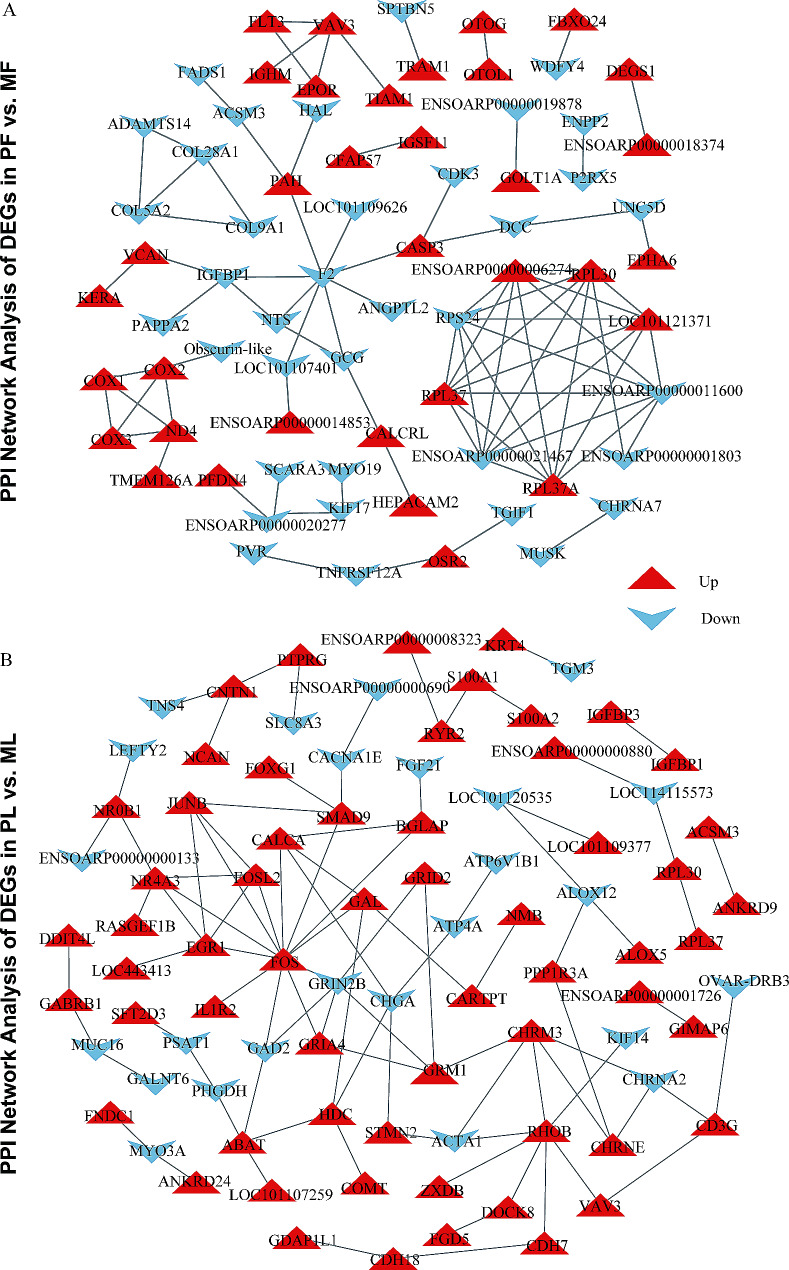
PPI network analysis of DEGs. STRING-based PPI network analyses of DEGs in PF vs. MF (**A**) and PL vs. ML (**B**). This graph used different shapes with different colors to represent the up-regulation and down-regulation of genes (proteins)

The DEGs of the two periods were separately analyzed by using the STRING database [36]. The protein-protein interaction (PPI) network contained 85 protein-protein pairs in PF vs. MF (Fig. 7A) and 93 pairs in PL vs. ML (Fig. 7B). We used the cytoHubba plug-in in Cytoscape to screen the TOP10 core genes (proteins). In the PF vs. MF group, the TOP10 core genes (proteins) were LOC101121371, RPL37A (Ribosomal Protein L37a), ENSOARP00000021467, RPL37 (Ribosomal Protein L37), RPS24 (Ribosomal Protein S24), ENSOARP00000006274, RPL30 (Ribosomal Protein L30), ENSOARP00000011600, F2 (Coagulation Factor II), COX2 (Cytochrome C Oxidase Polypeptide II). In the PL vs. ML group, the TOP10 core genes (proteins) were FOS (Fos Proto-Oncogene), EGR1 (Early Growth Response 1), FOSL2 (FOS Like 2), GRIN2B (Glutamate Ionotropic Receptor NMDA Type Subunit 2B), NR4A3 (Nuclear Receptor Subfamily 4 Group A Member 3), JUNB (JunB Proto-Oncogene), RHOB (Ras Homolog Family Member B), GRM1 (Glutamate Metabotropic Receptor 1), CALCA (Calcitonin Related Polypeptide Alpha), CHRM3 (Cholinergic Receptor Muscarinic 3). We focused on previously selected DEGs and their interacting proteins. For DEGs common to both phases, IGFBP1 has four interacting proteins (VCAN (Versican), PAPPA2 (Pappalysin 2), F2, NTS (Neurotensin)) in the follicular phase and only one (IGFBP3 (Insulin Like Growth Factor Binding Protein 3)) in the luteal phase; VAV3 has four interacting proteins (TIAM1 (T-Lymphoma Invasion And Metastasis-Inducing Protein 1), FLT3 (Fms Related Receptor Tyrosine Kinase 3), EPOR (Erythropoietin Receptor), IGHM (Immunoglobulin Heavy Constant Mu)) in the follicular phase and two interacting proteins (CD3G (CD3 Gamma Subunit Of T-Cell Receptor Complex), RHOB (Ras Homolog Family Member B)) in the luteal phase. In addition, the analysis results showed that FNDC1 might interact with MYO3A (Myosin IIIA), and other DEGs we selected (GABRG1, TMEFF2) did not get protein-protein interaction pairs.

### Target gene prediction of lncRNAs and search for ceRNAs relationship pairs

**Fig. 8:**
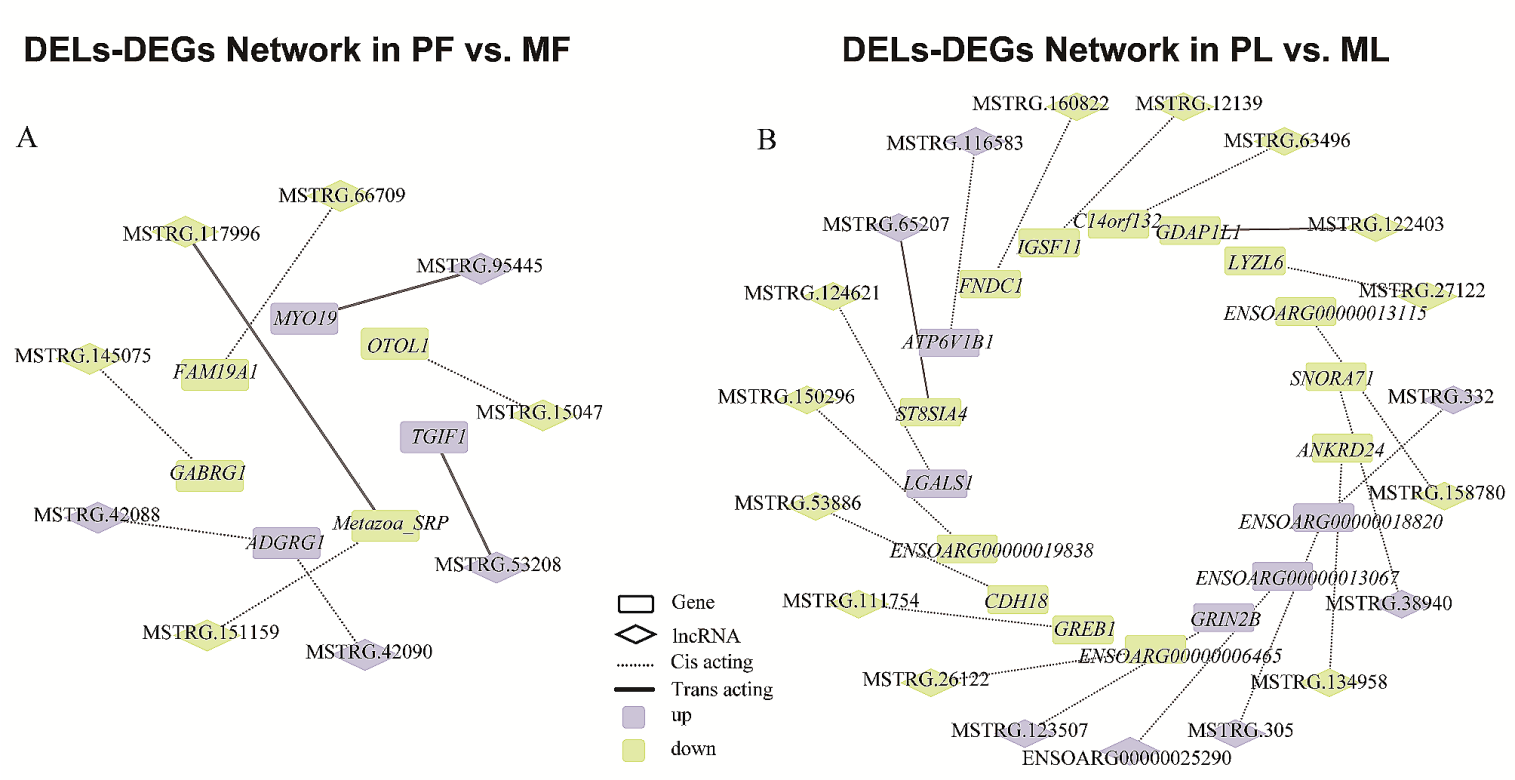
The network of DELs interaction with DEGs. Among all the predicted targeting relationships, only the relationship pairs in which both the lncRNAs and the target genes are differentially expressed are shown. There are nine pairs obtained in PF vs. MF (**A**) and twenty pairs in PL vs. ML (**B**)

By calculating the expression correlation and positional relationship between lncRNAs and genes, we obtained the prediction results of lncRNA target genes. A total of 1322 target relationship pairs (551 pairs in PF vs. MF and 771 pairs in PL vs. ML) were obtained for the target gene prediction of DELs (Supplementary Table S7). Among these predicted relationships, we only retained the relationship pairs in which both the lncRNAs and the target genes were differentially expressed. Finally, nine pairs (six cis-acting pairs and three trans-acting pairs) were obtained in the follicular phase (Fig. 8A), and twenty pairs (seventeen cis-acting pairs and two trans-acting pairs) in the luteal phase (Fig. 8B). *GABRG1*, a DEG that was trans-regulated by MSTRG.145,075, and *FNDC1* was trans-regulated by MSTRG.160,822. These two genes with a large differential expression trend were previously selected.

As one of the specific implementation forms of trans-acting, the ceRNA network relationship of lncRNA-miRNA-gene has been extensively studied and a large number of regulatory networks have been found. To identify possible ceRNA regulatory networks in the five trans-acting pairs we obtained, all known mature sequences of sheep miRNAs from the miRBase database [37] were used to construct the potential networks. Due to the negative correlation coefficient, MSTRG.65,207-ST8SIA4 was excluded from the prediction, and only four pairs (MSTRG.53,208-*TGIF1*, MSTRG.95,445-*MYO19* (Myosin Head Domain Containing 1), MSTRG.117,996-*Metazoa_SRP*, MSTRG.122,403-*GDAP1L1* (Ganglioside Induced Differentiation Associated Protein 1 Like 1)) remained in the end. Software miRanda (v3.3a) [38] was used to evaluate the binding capacity of lncRNAs and mRNAs in these relationship pairs to all known miRNAs in sheep under the parameter conditions of “-sc 150” and “-en-15”. Finally, all prediction results were visualized with Cytoscape (v3.9.1) [39] (Supplementary Figure S2). We obtained only one potential ceRNA regulatory network: MSTRG.53,208-oar-miR-329b-5p-*TGIF1*.

## Discussion

The pituitary is the most important endocrine gland, acting on various stages of the reproductive process through a variety of hormones. FSH and LH synthesized and secreted by the adenohypophysis affect ovulation [40], and oxytocin (OTX) stored and released by the neurohypophysis regulates childbirth [41]. In the study of the reproductive process, the pituitary cannot be bypassed. There have been studies on the expression profiles of pituitary mRNA and lncRNA in sheep with different fertility. The samples used in this research had *FecB* mutations, including *FecB* BB vs. *FecB* B + Hu sheep and *FecB* BB vs. *FecB* + + Small Tail Han sheep [23, 42]. However, our previous research found that there are still high-yielding individuals even in *FecB* + + Small Tail Han sheep. This phenomenon aroused our research interest, and we carried out transcriptomics research at the pituitary level of different fecundity groups of *FecB* + + Small Tail Han sheep.

We performed GO and KEGG enrichment analysis on the DEGs screened under the conditions of fold change > 1.6 and *P* < 0.05. The DEGs in the two periods were co-enriched in some GO items such as calcium ion binding. Ca^2+^ is a universal second messenger [43]. The formation of synaptic activity, the secretion of various molecules, and the transcriptional regulation of many genes are all affected by Ca^2+^ [44]. Ca^2+^ plays an important role in both male and female reproduction [45]. In addition, the differential genes in the two periods were co-enriched in KEGG items such as the cAMP signaling pathway, which was also shown to affect the reproductive process [46, 47]. Interestingly, the Ca^2+^ and cAMP pathways are the major signaling systems controlling almost all secretory gland functions in secretory epithelial cells, and there is a complex interplay between them [48].

To further identify potential key genes, we increased the screening criteria to reduce the number of target genes. After retrieving the functions of the remaining genes one by one, some genes with potentially important roles were finally retained. In addition, we also paid attention to the genes that were differentially expressed in the follicular phase and the luteal phase comparison group, as well as the genes with potential lncRNA-Gene regulatory relationship.

IGFBP1 was usually shown to inhibit the action of IGF (Insulin Like Growth Factor). This process was achieved by competitively inhibiting the combination of IGF and IGFR (Insulin Like Growth Factor 1 Receptor) [49]. The IGF system has been widely documented to affect animal reproduction [50, 51]. In vitro experiments at the pituitary level showed that exogenous IGF-1 can increase the secretion of LH in sheep pituitary cells [52]. The low level of LH in the breeding season of nutrient-deficient ewes was related to the content of IGFBP in the pituitary and circulatory systems [53]. IGFBP was found to be a potential regulator of gonadotropin function in the study of IGF-1 and IGFBP in the pituitary and circulatory systems of cows in different estrual periods [54]. The activity of IGFBP fluctuated in the pituitary and was associated with changes in the phase of the estrous cycle [55]. During this process, IGFBP regulated IGF-I and ultimately affected the release of gonadotropins. The different expression trends of *IGFBP1* in the follicular and luteal phases in our study also suggest the volatility of this regulation. In addition, PPI network analysis also provided evidence for the involvement of IGFBP1 in the reproductive process. PAPPA2, which interacted with IGFBP1 in the follicular phase, was involved in IGF signaling and identified as a candidate gene affecting fecundity in sheep [56, 57]. IGFBP3 interacts with IGFBP1 during the luteal phase.

*VAV3* is another gene that was differentially expressed in both periods. By regulating the transition between GTP and GDP in GTPases family proteins, VAV3 activates GTPases Rho protein, thus causing a series of reactions in downstream signaling pathways [58]. In studies of prostate and breast cancer, *VAV3* acted as an oncogene to activate androgen receptors and estrogen receptors to promote the growth of cancer cells [59, 60]. Although its function in the pituitary was not characterized, VAV3 has a potential function in response to androgen and estrogen [61]. That is to say, the high expression of *VAV3* in the two periods showed a stronger response of the pituitary in the high-yielding group to androgen and estrogen.

*GABRG1* encoded a gamma subunit of the GABAA receptor (gamma-aminobutyric acid type A receptor) [62]. The GABA system usually acts as an inhibitory neurotransmitter to regulate the activities of the central nervous system [63]. Researchers have also pointed out that GABAR could bind steroids, including peripheral synthetic and neurosteroids [64]. Premenstrual dysphoric disorder was a serious mood disorder during female reproduction. Research has pointed out that the interaction of hormone fluctuations (including estradiol, progesterone, and progesterone metabolites) and γ-aminobutyric acid type A receptors were involved in this process. This demonstrates the ability of GABRG1 to respond to steroid hormones and affect reproductive processes [65, 66]. In addition, we also predicted that MSTRG.145,075 was a cis-acting element of *GABRG1*, regulating the expression of *GABRG1*.

FNDC1, a member of the fibronectin type III domain-containing (FNDC) protein family, was commonly used as a prognostic biomarker for specific cancers [67]. In a study on prostate cancer, miR-1207-3p regulated the androgen receptor via FNDC1/fibronectin, demonstrating the promoting effect of FNDC1 on androgen receptor expression [68]. *FNDC1* was predicted as a candidate indicator of pregnancy status in cows, and a link between *FNDC1* and the reproductive process was also demonstrated [69]. MSTRG.160,822 was predicted to be a cis-acting element of *FNDC1*, regulating its expression.

Based on the analysis results and gene functions, we proposed the following hypotheses. First, the pituitary receives steroid hormone signals from the ovary and uterus, and *VAV3*, *GABRG1*, and *FNDC1* played a role in this process and then regulate the reproductive process through gonadotropins. *IGFBP1* was directly involved in the regulation of gonadotropin secretion, and ultimately affected litter size.

Some studies have pointed out that the *FecB* gene mutation increased sheep fecundity due to the inhibition of the BMP/SMAD pathway [70, 71]. Interestingly, the two genes we screened (*TGIF1* and *TMEFF2*) compensated for the effect of the *FecB* mutation in different periods. TGIF1 inhibits TGFβ signaling by directly binding to SMAD2 (SMAD Family Member 2) and SMAD4 (SMAD Family Member 4) complexes and inhibiting SMAD-mediated transcription [72]. Existing studies have pointed out that the site of *TGIF1* (g.37,866,222 C > T) was related to the number of offspring born in Small Tail Han sheep [73]. In addition, MSTRG.53,208 was predicted to be a trans-acting factor of *TGIF1*. After predicting the ability to bind to all known miRNAs, we identified a possible ceRNA regulatory relationship: MSTRG.53,208-oar-miR-329b-5p-*TGIF1*. Since we did not obtain expression profiles of miRNAs, the construction of the ceRNA network used all known miRNAs in sheep in the miRBase database. MSTRG.53,208 was a new lncRNA we obtained, which has not been reported before. Therefore, the regulatory relationship between MSTRG.53,208 and oar-miR-329b-5p was based on the prediction results of the ceRNA regulatory mechanism and sequence complementarity. These results still need further verification. *TMEFF2* encodes an EGF-like and two follistatin-like domains [74]. The follistatin-like domain can act as an endogenous blocker of the TGF-β signaling pathway [75, 76]. In the study of the effect of *FecB* mutation on DNA methylation in Small Tail Han sheep ovaries, *TMEFF2* was identified as a gene related to female reproduction [77]. In conclusion, although our research population has no *FecB* mutation, there are still differences in fecundity. We speculated that these genes compensate for the effect of *FecB* mutation.

## Conclusions

Transcriptome analysis revealed mRNA and lncRNA expression profiles in follicular and luteal stages of Small Tail Han sheep with different fertility without *FecB* mutations. Through gene functional analysis, we identified some key genes and their regulatory relationships. They regulate the reproductive process by affecting the pituitary response to steroid hormones and the release of gonadotropins. Some genes compensate for the effect of *FecB* mutation. These results provide new insight into the mechanism of high fecundity in Small Tail Han sheep without *FecB* mutation.

## Materials and methods

### Ethical consideration and samples collection

In this study, the handling of all experimental animals was approved by the Animal Ethics Committee of the Institute of Animal Science, Chinese Academy of Agricultural Sciences (No. IASCAAS-AE-03, 12 December 2016).

Based on TaqMan assays, 142 *FecB* + + Small Tail Han sheep were selected from a core herd of Shandong Province, China. Then twelve of them were selected according to lambing records (six polytocous sheep and six monotocous sheep) and physical condition (same body condition) for subsequent experiments. All selected ewes were bred in Tianjin (117.2 E, 39.13 N) with full consideration of animal welfare. Experimental animals were treated with vaginal sponges (InterAg Co., Ltd., New Zealand) (progesterone 300 mg, placed twelve days), and the removal time was set as 0 h. Refer to previous research, six ewes (three polytocous sheep and three monotocous sheep) were euthanized at 48 h (follicular phase), and six ewes (three polytocous sheep and three monotocous sheep) at 216 h (luteal phase) [78, 79]. After euthanasia, pituitary samples were collected and preserved as soon as possible for subsequent analysis. In summary, we obtained two comparison groups, and subsequent analysis will be carried out on this basis. These groups were polytocous sheep in the follicular phase (PF) vs. monotocous sheep in the follicular phase (MF) and polytocous sheep in the luteal phase (PL) vs. monotocous sheep in the luteal phase (ML). This nomenclature was primarily based on the lambing records of the experimental group and the observation of ovulation by laparoscopic observation [80]. Meanwhile, it was consistent with previous research on this group [13, 78, 79].

### RNA library construction, sequencing, and data processing

According to the requirements of the manual, the total RNA of twelve pituitary samples was extracted by using the TRIzol Reagent (Invitrogen, Carlsbad, CA, USA). Then, a NanoDropTM 2000 (Thermo ScientificTM, Wilmington, DE, USA) instrument was used to measure the purity (OD 260/280: 1.8–2.0) and concentration of RNA samples. RNA integrity (RNA Integrity Number > 7) was assessed by using an Agilent 2100 System (Agilent Technologies, Santa Clara, CA, USA). RNA samples that pass the quality test will be used for RNA-Seq.

Total RNA (three µg) was used for generating cDNA libraries. The ribosomal RNA (rRNA) was removed by Epicentre Ribo-ZeroTM rRNA Removal Kit (Epicentre, Madison, WI, USA). The libraries were constructed following the instructions of the NEB Next Ultra Directional RNA Library Prep Kit for Illumina (NEB, Ipswich, MA, USA). Finally, the pooled library was sequenced by Hiseq X (Illumina, San Diego, CA, United States).

The raw data were obtained by converting the original image files generated by sequencing into sequence files by bcl2fastq (v2.17.1.14) software. Raw reads were filtered to ensure the quality of further analytical data by using in-house Perl scripts (Annoroad Gene Technology Co., Ltd, Beijing, China). The clean data was obtained after filtering, and statistics analyses were performed on its quantity and quality, including Q30 statistics, data quantity statistics, base content statistics, etc.

The sheep reference genome (Oar v.3.1) was downloaded from ENSEMBL. The clean reads were aligned by using HISAT2 (v.2.0.5) [81] with the parameters “-rna-strandness RF” and “-dta -t -p 4”. Then the software String Tie (v1.3.2d) [82] was used to assemble with the parameters “-G ref.gtf -rf -1”.

### LncRNAs and mRNAs identification and differential expression analysis

Through the above steps, known lncRNA and mRNA have been identified based on the annotations of the reference genome. To identify new lncRNAs, some basic screening conditions were set, and various software were used to predict the coding potential. In general, after removing known mRNAs and other non-coding RNAs of the species, new lncRNA transcripts should also meet the following conditions: its reads coverage is > five, the number of exons is ≥ two, and its length is ≥ 200 bp. More importantly, new lncRNAs need to pass the screening of several coding potential prediction software such as CNCI [30], CPC [31], PFAM [32], and CPAT [33]. The CNCI with the parameters “-score 0 -length 199 -exon_num 2” distinguished the coding and non-coding potential of sequences based on features of adjacent nucleotide triplets. The CPC compares the transcript with the known protein database by Blastx and evaluates the coding potential of the transcript through the classifier of the support vector machine. For CPC and CNCI, transcripts were considered non-coding if the sequence had a score < 0. Transcripts are considered non-coding if the sequence differs from known protein domain transcripts, which is the predictive principle of PFAM. In the results, if E-value < 0.001, the sequence was considered to have encoding potential. The parameters of PFAM were set as “minimum protein length: 60 and others as the default”. The CPAT constructed a logistic regression model by analyzing ORF features and calculating Fickett and Hexamer scores to judge coding potential. Finally, a collection of new lncRNAs was obtained for further analysis.

The HTSeq python package (v0.6.0) [83] with parameters “-i gene_id -f bam -s reverse -a 10 -q” was adopted to calculate read counts. Fragments per kilobase of transcript per million mapped reads (FPKM) represented the level of gene expression. To obtain DELs and DEGs, DESeq2 [84] was adopted to screen with fold change > 1.6 and *P* < 0.05 as the threshold. To explore the relationship between different libraries, based on the log_10_(FPKM) value of each gene and lncRNA, a systematic clustering analysis was performed using the Euclidean distance method.

### Bioinformatics analysis of DEGs

GO and KEGG are commonly used for the functional enrichment of gene sets to explain the potential function of genes. We performed GO and KEGG analysis using the online software DAVID [34] and KOBAS 3.0 [35], respectively. A threshold of *P* < 0.05 was used as a criterion for the determination of whether the enrichment analysis was significant.

The STRING [85] is a protein interaction network database based on public databases and literature information. This database was used to analyze the interactions between DEGs. The mode selected for the analysis was “Multiple Proteins”, the species was “*Ovis aries*”, only proteins with interaction relationships were displayed. Settings options are provided in the String database. We set the minimum required interaction score option to medium confidence (0.400). Under this condition, the optimal amount of protein was retained. Using this tool, we obtained the protein-protein interaction (PPI) network between proteins encoded by DEGs. In addition, Cytoscape (v3.9.1) [39] was used to beautify the PPI network diagram, and the cytoHubba plug-in was used to screen the top 10 core genes (proteins) under default parameters.

### Bioinformatics analysis of DELs

LncRNAs act on protein-coding genes through cis-acting or trans-acting to realize their functions. Among them, lncRNAs located 50 kb upstream and downstream of protein-coding genes were identified as potential cis-elements, and lncRNAs with a correlation coefficient of ≥ 0.9 with protein-coding gene expression were identified as a potential trans-element. Then a lncRNAs-mRNAs network based on the targeting relationship was built and visualized using Cytoscape (v3.9.1) [39].

In the trans-acting type, lncRNAs can regulate microRNAs (miRNAs) activities through base-pairing interactions [86]. Then miRNAs can act on mRNAs to regulate gene expression. This mode of action was known as the competing endogenous RNAs (ceRNAs) mechanism. To determine the ceRNAs relationship network, predictions were made using the obtained trans-action relationship pairs and the miRNAs information in the database. Firstly, the mature sequences of all known miRNAs in sheep were obtained in the miRBase database [37]. Prediction of the binding capacity of lncRNAs and miRNAs using miRanda (v3.3a) [38]. The input files were lncRNAs sequences and miRNAs sequences, and the parameters were set to “-sc 150” and “-en -15”. The same parameters were used to assess the binding capacity of miRNAs and mRNAs. The binding ability of the mRNA in the trans-regulatory relationship pair to all known miRNA mature sequences was predicted. Finally, according to the predicted results, the ceRNAs relationship network in the trans-acting relationship was obtained.

### RT-qPCR validation

To verify the accuracy of RNA sequencing, six mRNAs and six lncRNAs were selected for the reverse-transcription quantitative polymerase chain reaction (RT-qPCR). Primers were designed by Primer Premier 6 and synthesized by Sangon Biotech (Shanghai, China). The internal reference gene was β-actin (Supplementary Table S8). The reverse transcription program and quantification program were performed according to the instructions of PrimeScript™ RT reagent Kit (Takara, Beijing, China) and TB Green® Premix Ex Taq II (Takara, Beijing, China), respectively. Relative quantification of expression was compared to the internal reference gene and analyzed using the 2^−ΔΔCt^ method.

### Electronic supplementary material

Below is the link to the electronic supplementary material.

Supplementary Material 1

Supplementary Material 2

Supplementary Material 3

Supplementary Material 4

Supplementary Material 5

Supplementary Material 6

Supplementary Material 7

Supplementary Material 8

Supplementary Material 9

Supplementary Material 10

## Data Availability

All the RNA-seq reads have been deposited in the Sequence Read Archive (https://www.ncbi.nlm.nih.gov/sra) with the accession codes (BioProject ID: PRJNA792697).

## Abbreviations

FSH: Follicle-stimulating hormone
LH: Luteinizing hormone
FecB: Fecundity Booroola
PF: Polytocous sheep in the follicular phase
PL: Polytocous sheep in the luteal phase
MF: Monotocous sheep in the follicular phase
ML: Monotocous sheep in the luteal phase
DEGs: Differentially expressed genes
DELs: Differentially expressed lncRNAs
ceRNA: competing endogenous RNA
VAV3: Vav Guanine Nucleotide Exchange Factor 3
GABRG1: Gamma-Aminobutyric Acid A Receptor, Gamma 1
FNDC1: Fibronectin Type III Domain Containing 1
IGFBP1: Insulin-like Growth Factor Binding Protein 1
TGIF1: Transforming Growth Factor-Beta-Induced Factor1
TMEFF2: Transmembrane Protein With EGF Like And Two Follistatin Like Domains 2
BMPR1B: Bone Morphogenetic Protein Receptor Type 1B
GnRH: Gonadotropin-releasing hormone
TGFB1: Transforming Growth Factor Beta 1
HSD17B12: Hydroxysteroid 17-Beta Dehydrogenase 12
lncRNAs: long non-coding RNAs
CNCI: Coding-Noncoding Index
CPC: Coding Potential Calculator
PFAM: Protein families database
CPAT: Coding Potential Assessment Tool
RT-qPCR: Real-time quantitative polymerase chain reaction
GO: Gene Ontology
KEGG: Kyoto Encyclopedia of Genes and Genomes
PPI: protein-protein interaction
RPL37A: Ribosomal Protein L37a
RPL37: Ribosomal Protein L37
RPS24: Ribosomal Protein S24
RPL30: Ribosomal Protein L30
F2: Coagulation Factor II
COX2: Cytochrome C Oxidase Polypeptide II
FOS: Fos Proto-Oncogene
EGR1: Early Growth Response 1
FOSL2: FOS Like 2
GRIN2B: Glutamate Ionotropic Receptor NMDA Type Subunit 2B
NR4A3: Nuclear Receptor Subfamily 4 Group A Member 3
JUNB: JunB Proto-Oncogene
RHOB: Ras Homolog Family Member B
GRM1: Glutamate Metabotropic Receptor 1
CALCA: Calcitonin Related Polypeptide Alpha
CHRM3: Cholinergic Receptor Muscarinic 3
VCAN: Versican; PAPPA2:Pappalysin 2
NTS: Neurotensin
IGFBP3: Insulin Like Growth Factor Binding Protein 3
TIAM1: T-Lymphoma Invasion And Metastasis-Inducing Protein 1
FLT3: Fms Related Receptor Tyrosine Kinase 3
EPOR: Erythropoietin Receptor
IGHM: Immunoglobulin Heavy Constant Mu
CD3G: CD3 Gamma Subunit Of T-Cell Receptor Complex
RHOB: Ras Homolog Family Member B
MYO3A: Myosin IIIA
MYO19: Myosin Head Domain Containing 1
GDAP1L1: Ganglioside Induced Differentiation Associated Protein 1 Like 1
OTX: oxytocin
IGF: Insulin Like Growth Factor
IGFR: Insulin Like Growth Factor 1 Receptor
SMAD2: SMAD Family Member 2
SMAD4: SMAD Family Member 4
FPKM: Fragments Per Kilobase per Million
