# Supplementary figures and images for: Key mRNAs and lncRNAs of pituitary that affect the reproduction of FecB + + small tail han sheep
Source: BMC Genomics. 2024 Apr 22;25:392. doi: 10.1186/s12864-024-10191-8 (PMC11034058; doi:10.1186/s12864-024-10191-8)

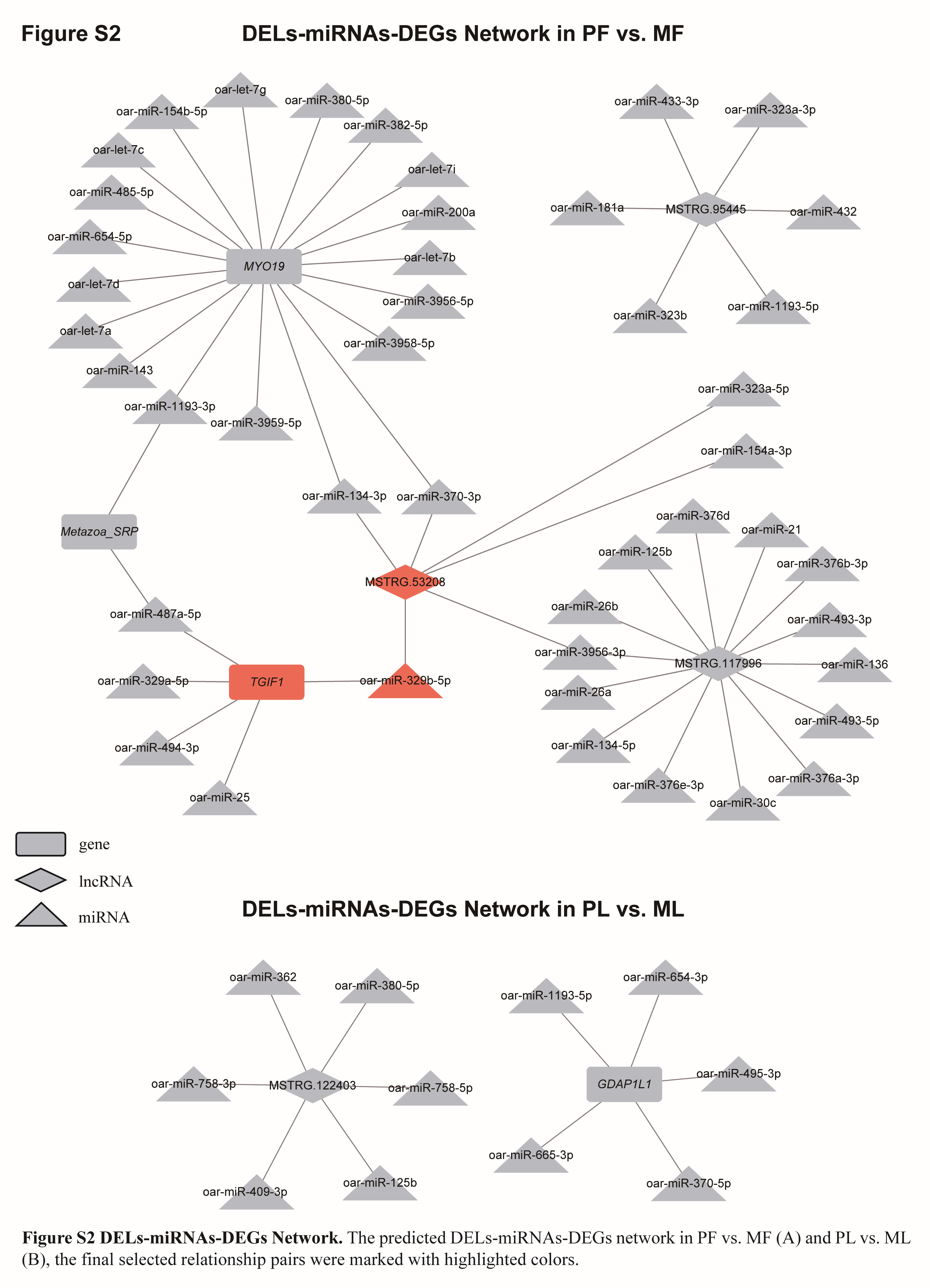

Supplement: Supplementary file 10 — Supplementary Material 10 [file 12864_2024_10191_MOESM10_ESM.tiff]
